# Spheroid-Based 3D Models to Decode Cell Function and Matrix Effectors in Breast Cancer

**DOI:** 10.3390/cancers17213512

**Published:** 2025-10-31

**Authors:** Sylvia Mangani, Christos Koutsakis, Nikolaos E. Koletsis, Zoi Piperigkou, Marco Franchi, Martin Götte, Nikos K. Karamanos

**Affiliations:** 1 Biochemistry, Biochemical Analysis & Matrix Pathobiology Research Group, Laboratory of Biochemistry, Department of Chemistry, University of Patras, 26504 Patras, Greece; ckoutsakis@upatras.gr (C.K.); nkoletsis@uptras.gr (N.E.K.); zoipip@upatras.gr (Z.P.); 2 Department for Life Quality Studies, University of Bologna, 47921 Rimini, Italy; marco.franchi3@unibo.it; 3 Department of Gynecology and Obstetrics, Münster University Hospital, 48149 Münster, Germany; martingotte@uni-muenster.de

**Keywords:** extracellular matrix, 3D breast cancer cell models, spheroids, functional cell properties, matrix metalloproteinases, syndecans

## Abstract

**Simple Summary:**

Traditional two-dimensional (2D) cell cultures are widely used in cancer research but fail to replicate the complex interactions that occur in tumors in vivo. Three-dimensional (3D) models more closely mimic the tumor microenvironment by preserving dynamic cell–matrix communication. In this study, we developed and characterized 3D spheroids from two breast cancer cell lines with distinct metastatic potential (MCF-7 and MDA-MB-231). Significant differences in cell morphology, epithelial-to-mesenchymal transition (EMT) markers expression, and functional behavior compared to 2D cultures were observed. The spheroids also showed the distinct expression of key receptors (*ERs, EGFR, IGF1R*) and matrix molecules (syndecans and matrix metalloproteinases). Bioinformatic analysis further revealed the clinical relevance of these matrix regulators in breast cancer prognosis. Overall, these findings introduce an informative, matrix-free cell culture platform for studying breast cancer progression and potentially exploring new therapeutic approaches.

**Abstract:**

**Background/Objectives**: Conventional two-dimensional (2D) cell cultures offer valuable insights into cancer cell biology; however, they lack in replicating the complex interactions present in solid tumors. Therefore, research has shifted towards the development of three-dimensional (3D) cell models that recapitulate the dynamic cell–cell and cell–matrix interactions within the complex tumor microenvironment (TME), better resembling tumor growth and initial stages of dissemination. Extracellular matrix, a key component within the TME, regulates cell morphology and signaling, influencing key functional properties. Breast cancer remains the most frequently diagnosed cancer type in women and a leading cause of cancer-related mortality. **Methods**: The aim of the present study was the development of breast cancer cell-derived spheroids, utilizing two breast cancer cell lines with differential estrogen receptor (ER) expression profile, and their characterization in terms of morphology, functional properties, and expression of epithelial-to-mesenchymal transition (EMT) markers and matrix signatures implicated in breast cancer progression. To this end, the ERα-positive MCF-7, and ERβ-positive MDA-MB-231 breast cancer cell lines were utilized. **Results**: Our findings revealed notable phenotypic transitions between 2D and 3D cultures, which were further supported by differential EMT markers expression. Moreover, spheroids exhibited distinct expression profiles of key receptors [*ERs*, epidermal growth factor receptor (*EGFR*) and insulin-like growth factor receptor (*IGF1R*)] and matrix molecules (syndecans, and matrix metalloproteinases), accompanied by altered functional cell properties. Bioinformatic tools further emphasized the interplay between the studied matrix regulators and their prognostic relevance in breast cancer. **Conclusions**: Overall, this study introduces a simple yet informative 3D breast cancer model that captures key TME features to better predict cell behavior in vitro.

## 1. Introduction

Conventional two-dimensional (2D) cell cultures are widely used in cancer research, due to their simplicity, low cost, and ability to provide key insights and valuable information regarding molecular characteristics and cell functional properties [1]. Cells grown in monolayers exhibit uniform access to nutrients, O_2_, and therapeutic agents, which, however, do not reflect the in vivo microenvironment of solid tumors [2,3]. This limitation has driven research to the development of advanced three-dimensional (3D) cell culture models, which more accurately mimic the dynamic interactions being present in in vivo conditions, providing a more representative platform for cancer research [4]. Emerging 3D cell culture platforms, particularly cancer cell-derived spheroids, offer a more realistic representation of solid tumor properties by capturing the complex cell–cell and cell–matrix interactions within the tumor microenvironment (TME), which hold a critical role in cancer development and progression [5].

Breast cancer constitutes a highly heterogeneous disease and remains the leading cause of cancer-related mortality among women worldwide [6]. Among breast cancer subtypes, Luminal A (ER+ and/or PR+, HER2-) represents approximately 70% of all cases and is mostly associated with better patient prognosis [7,8]. On the other hand, triple-negative breast cancer (TNBC) (ER-, PR-, HER2-) is the most aggressive subtype appearing in 15–20% of cases, and is often diagnosed at later stages with poor patient outcomes [7,9]. In breast cancer, 17β-estradiol (E2)-ER signaling regulates critical functional properties, such as cell differentiation, proliferation, epithelial-to-mesenchymal transition (EMT), migration, invasion, metastasis, and resistance to treatment. Furthermore, the non-genomic ER-mediated E2 effects result in the rapid activation of receptor tyrosine kinases (RTKs) and downstream signaling pathways [10].

The coordinated actions of ERs and extracellular matrix (ECM) molecules mediate matrix remodeling within the TME [10]. ECM constitutes a complex 3D dynamic intercellular macromolecular network that offers structural support to the cells within the tissues, while also holding significant functional roles in driving cell morphology, signaling, and cellular functional properties. Various ECM molecules, including glycosaminoglycans, proteoglycans (PGs), receptors/growth factor (GF) receptors, and enzymes, such as matrix metalloproteinases (MMPs), exhibit critical roles in oncogenesis [11]. Notably, ECM composition and mechanical properties regulate EMT, endowing cancer cells with invasive/migratory potential and stemness characteristics [12,13,14].

Within the TME, the interplay between stromal and cancer cells creates a provisional matrix that mediates cancer cell reprogramming and induces EMT, fostering aggressive and invasive characteristics [15,16]. Matrix remodeling, primarily driven by MMPs, further generates pathways that enable cancer cells to migrate and invade the aligned and stiff ECM [17]. ECM further facilitates the storage and controlled presentation of signaling molecules to neighboring cells, thereby inducing signaling pathways that influence cell fate [18]. Among RTKs, the EGFR and the IGF-IR mediate signaling pathways that regulate the expression of target genes during breast cancer progression, thereby influencing cell proliferation, metabolic reprogramming, apoptotic evasion, EMT, invasion, and metastasis [19,20].

Among the critical ECM components mediating cell signaling are syndecans (SDCs), a family of four transmembrane heparan sulfate PGs in mammals [21]. Their aberrant expression is linked to cancer progression, particularly influencing prognosis and clinical outcomes, and mediating resistance to therapy [15,22]. E2-ER signaling can modulate the expression of SDCs in both ERα+ and ERα- breast cancer cells [22]. Furthermore, E2-ERα signaling drives MMP-2, MMP-9, and MT1-MMP upregulation, promoting ECM remodeling and thereby enhancing breast cancer cell invasion and migration, while ERβ has also been found to regulate the expression of MMP-2 and MMP-7 [10].

Although the regulatory role of ECM components in cancer initiation and progression has been established using 2D cancer cell cultures, limited studies have revealed their contribution within 3D breast cancer cell models to our knowledge. Notably, Koedoot et al. (2021) have previously shown–using RNA-seq analysis–the differential gene expression in 2D vs. 3D breast cancer cell lines, particularly in regard to matrix organization [23]. Furthermore, recent studies from our research group have revealed valuable insights into ECM dynamics and epigenetic regulation in 3D breast cancer cell models [24,25,26]. The aim of the present study was, therefore, to develop breast cancer cell-derived spheroids (utilizing breast cancer cell lines with distinct ER expression profile and metastatic potential) in a matrix-free cell culture platform, and characterize them in terms of morphological features, functional properties, and expression of critical EMT markers, receptors, and major ECM components implicated in breast cancer progression.

## 2. Materials and Methods

### 2.1. Cell Cultures and Reagents

MCF-7 (Luminal A; ERα+; low-metastatic potential; epithelial morphology in 2D culture) and MDA-MB-231 (TNBC; ERα-/ERβ+; high-metastatic potential; mesenchymal-like morphology in 2D culture) breast cancer cell lines were obtained from the American Type Culture Collection (HTB-22, HTB-26, ATCC, Manassas, VA, USA). Breast cancer cell lines were routinely cultured at 37 °C (in a humidified atmosphere of 5% CO_2_) in complete Dulbecco’s modified Eagle’s medium (DMEM; LM-D1110, Biosera, Cholet, France), supplemented with 10% fetal bovine serum (FBS; FB-1000, Biosera, Cholet, France), as well as with antimicrobial agents (100 IU/mL penicillin, 100 μg/mL streptomycin, 10 μg/mL gentamycin sulfate and 2.5 μg/mL amphotericin B), and 2 mM L-glutamine (Biosera, Cholet, France). Sub-cultivation was performed using trypsin-EDTA 1X in PBS (LM-T1706; Biosera, Cholet, France). Cell lines were routinely tested to confirm the absence of mycoplasma contamination.

### 2.2. Three-Dimensional Cell Cultures; Spheroids Development

Breast cancer cell-derived spheroids were developed by culturing cancer cells in U-shape, round bottom 96-well plates with ultra-low adhesive properties (911606, SPL Life Sciences, Pocheon-si, Gyeonggi-do, Republic of Korea). MCF-7 and MDA-MB-231 cells were seeded at a density of 5000–15,000 cells per well, depending on the experimental requirements, and incubated in complete medium (DMEM 10% FBS), allowing for cells self-aggregation and spheroid development. Spheroid growth was monitored through a phase-contrast microscope (CKX41, Evident Scientific, Hamburg, Germany) with an integrated digital camera (QImaging MicroPublisher 3.3RTV, Surrey, BC, Canada) to determine the optimal time point for downstream analyses (72 h of culture was sufficient for spheroid development). The medium was then discarded, and cells were cultured following a 16–20 h serum-free medium starvation period. Subsequent analyses included their morphological characterization by scanning electron microscopy. Following that, spheroids were either collected for total RNA extraction, or transferred to standard, flat-bottom well plates, to examine their dissemination and migratory properties.

### 2.3. Scanning Electron Microscopy Imaging

For detailed analysis of the morphological features of 3D spheroids, scanning electron microscopy (SEM) was utilized. Once breast cancer cell-derived spheroids were formed, they were gently washed with phosphate buffer (0.2 M and pH 7.4). Afterwards, spheroids were fixed in Karnovsky’s solution [4% (*w*/*v*) PFA, 5% (*v*/*v*) glutaraldehyde, 0.04 M phosphate buffer solution] for 1 h at room temperature, to preserve cellular and structural integrity. Following fixation, they were washed with 0.1% cacodylate buffer and then post-fixed in 1% OsO_4_ in cacodylate buffer for 20 min. Next, spheroids were dehydrated with increasing concentrations of ethanol and subsequently subjected to critical point drying. The samples were mounted on a stub and then coated with a 5 nm palladium gold film using an Emitech 550 sputter-coater (Richmond Scientific, Chorley, UK). The 3D spheroids were finally observed and examined using a Philips 515 SEM (Eindhoven, The Netherlands) operated in secondary-electron mode to capture detailed surface morphology.

### 2.4. RNA Isolation, Reverse Transcription, and Real-Time qPCR Analysis

MCF-7 and MDA-MB-231 cells were cultured in 2D monolayers and 3D spheroids in complete medium for 72 h. Subsequently, complete medium was replaced with serum-free medium, and cells were serum-starved for 16–20 h. Total RNA was extracted using NucleoSpin^®^ RNA II Kit (Macherey-Nagel GmbH & Co. KG, Düren, Germany), according to the manufacturer’s protocol. RNA concentration was determined by measuring samples absorbance at 260 nm using a photometer (Implen GmbH, Munich, Germany), and its quality was further validated by assessing the 260/280 nm and 260/230 nm ratios. Total RNA from both 2D and 3D cultures was reverse transcribed using the PrimeScript™1st strand cDNA synthesis kit (Takara Bio Inc., Kusatsu, Japan). Real-time qPCR was performed according to the manufacturer’s instructions on a Rotor Gene Q system (Qiagen, Germantown, MD, USA). All reactions were performed in technical replicates, and a standard curve was included for each primers pair to confirm amplification efficiency. A melting curve analysis was further included to verify the specificity of the SYBR Green amplicons. The fluorescence threshold was set above the background to determine the threshold cycle (Ct) number, corresponding to the exponential amplification phase. Relative gene expression was calculated by the 2^−ΔΔCt^ method, normalizing the Ct values of target genes to the Ct of the housekeeping gene *ACTB*. Detailed information about the target genes, as well as the utilized primers, are listed in Table 1.

### 2.5. Immunofluorescence Staining and Imaging

MDA-MB-231 cells were seeded on sterile glass coverslips in a 24-well plate. Cells were cultured in complete medium up to 70–80% confluence, and then in the absence of serum 16–20 h. Next, cells were washed with PBS 1X and fixed in 4% (*w*/*v*) PFA in PBS 1X for 10 min, while, after fixation, three washes with PBS 1X were performed, followed by 1 h incubation at room temperature in blocking solution, consisting of 4% (*w*/*v*) BSA, 0.1% (*v*/*v*) Triton-X-100 in PBS 1X. The cells on the coverslips were subsequently incubated overnight with the primary antibody against E-cadherin (mouse, 1:100, Cell Signaling Technology, Danvers, MA, USA) at 4 °C, in a humidified environment. Following PBS 1X washes, the coverslips were incubated with the secondary antibody (goat anti-mouse Alexa Fluor-488, 1:500; Biotium, Fremont, CA, USA) for 1.5–2 h and three washes with PBS 1X were performed. The primary and secondary antibodies were diluted in 1% BSA/PBS-Tween 20 0.01% solution. Finally, the coverslips were mounted on microscope slides using the ProLong Gold Antifade mountant (ThermoFisher Scientific Inc., Waltham, MA, USA), which contains the dye DAPI (4,6-diamidino-2-phenylindole), that binds to the DNA of the cells, thus staining the nuclei in blue color. Visualization was performed using a fluorescent phase contrast microscope (CKX41, Evident Scientific, Hamburg, Germany) with an integrated digital camera (QImaging MicroPublisher 3.3RTV, Surrey, BC, Canada).

MCF-7 and MDA-MB-231 cells were seeded at a density of 10,000 cells per well in U-shape, round bottom 96-well plates with ultra-low adhesive properties. Cells were cultured in complete medium for 72 h, and then spheroids were serum-starved for 16–20 h. Afterwards, the immunofluorescence staining was performed, as described above. For the observation of the 3D spheroids, a confocal microscope (Leica TCS SP8, Wetzlar, Germany) was utilized.

### 2.6. Bioinformatic Tools

#### 2.6.1. Kaplan–Meier Plotter

The Kaplan–Meier plotter (https://kmplot.com/analysis/, accessed on 20 July 2025) was utilized to evaluate the prognostic value of specific gene expression levels in breast cancer subtypes. This bioinformatic tool integrates data from various public databases, including Gene Expression Omnibus (GEO), European-Genome-phenome Archine (EGA) and Cancer Genome Atlas (TCGA), providing comprehensive survival analyses based on gene expression profiles. The database offers survival analysis curves that illustrate the probability of patients’ survival over a defined time period, incorporating shorter intervals within the overall analysis. Thus, these plots allow for the estimation of survival rates in patients with different types of cancer, highlighting correlations between survival outcomes and the expression levels of individual genes (biomarkers) or combinations of genes [27]. OS analysis was performed with the following settings: [user selected probe set, split by auto select best cutoff, ER+/PR+ (IHC) status, *n* = 141] for Luminal A subtype patients, and [user selected probe set, split by auto select best cutoff, ER-/PR-/HER2- (IHC) status, *n* = 201] for TNBC subtype patients. 

#### 2.6.2. Interaction Networks Using the STRING Database

The STRING database (https://string-db.org/, accessed on 20 July 2025) is a bioinformatic platform for constructing and analyzing protein–protein interaction networks across any genome. The resulting network incorporates both direct physical interactions and indirect functional associations. Interaction data are integrated from diverse sources, including literature data mining, computational predictions based on co-expression and conserved genomic context, experimentally validated interactions, and known protein complexes or pathways. By combining multiple evidence types, STRING provides a comprehensive and robust view of protein networks, enabling the identification of overlapping cellular pathways and the exploration of their potential roles in complex biological processes [28]. Analysis was performed using the default settings, adding multiple proteins. 

#### 2.6.3. The Human Protein Atlas

The Human Protein Atlas, hereafter referred to as THPA (https://www.proteinatlas.org/, accessed on 25 July 2025), is a comprehensive database that examines the spatial distribution of proteins in tissues and cells, thus providing detailed information on their expression profiles at the cellular level. The atlas is organized into six main subcategories: (a) the Tissue Atlas, which maps proteins distribution across major human tissues and organs; (b) the Cell Atlas, detailing the subcellular localization of proteins; (c) the Pathology Atlas, examining how protein levels influence cancer patient survival; (d) the Blood Atlas, profiling protein expression in blood cells and secreted proteins; (e) the Brain Atlas, depicting protein distribution in human, mouse, and pig brains; and (f) the Metabolic Atlas, highlighting the roles of proteins in human metabolic pathways [29]. 

### 2.7. Spheroid Dissemination and In Vitro Wound Healing Assay

After 72 h of spheroid culture in complete medium, a 4 h-serum starvation period was implemented. Spheroids were then transferred individually (1 spheroid/well) to a standard, flat-bottom 96-well plate containing DMEM 5% FBS, to assess cancer cell dissemination. Representative images of cancer cell spreading were captured using a phase-contrast microscope equipped with a digital camera after 24 and 48 h, respectively. The spheroid core area was manually delineated and quantified based on contrast and cell density thresholds (ImageJ 1.50b Launcher Symmetry Software, LOCI; University of Wisconsin).

To assess the migratory capacity of the spheroid-derived cells, spheroids were transferred to a 48-well plate (containing DMEM 5% FBS, 5 spheroids per well), where they were allowed to spread until they covered the well surface. After 5 days for MCF-7 and 4 days for MDA-MB-231, the wound healing assay was conducted. To further evaluate the migratory capacity of 2D cell cultures, MCF-7 and MDA-MB-231 cells were seeded into 48-well plates and incubated in complete medium until a monolayer was formed. The medium was then replaced with serum-free medium, and cells were serum-starved overnight. The following day, a straight-line scratch was created in the cell monolayer using a sterile 10 μL pipette tip, and each well was washed 2–3 times with serum-free medium to remove any detached cells. Serum-free medium containing the cytostatic agent cytarabine (Sigma-Aldrich, Saint Louis, MO, USA, 10 μΜ) was then added to ensure that the observed wound closure actually reflects cell migration rather than proliferation [30]. The cytarabine concentration (10 µM) was chosen based on our research group’s previous studies, in which this concentration was experimentally validated to effectively inhibit cell proliferation without affecting cell migration in similar cell types [31]. After 40 min of incubation with cytarabine at 37 °C, representative images of the initial wound (0 h) were captured using a phase-contrast microscope equipped with a digital camera. Additional images were acquired after 24 h and 48 h, respectively, and cancer cell migration was assessed by quantifying the wound surface area (ImageJ software version 1.50b Launcher Symmetry Software, LOCI, University of Wisconsin, Madison, WI, USA). The analysis was based on average quantitative data from multiple measurements across several fields of the wounds, to ensure reliable and comparable results across conditions.

### 2.8. Statistical Analysis

Experiments were performed using three independent biological replicates. Within each biological replicate, technical replicates were also included to ensure reproducibility. Reported values are expressed as mean ± standard deviation (SD) of experiments in triplicate. Statistically significant differences were evaluated using two-tailed Student’s *t*-test to determine statistical differences between the two cell culture models. Differences were considered statistically significant at the level of *p* ≤ 0.05, *p* ≤ 0.01, and *p* ≤ 0.001 indicated by (*, **, ***) for the 3D cell model vs. 2D cell cultures comparison. Statistical analysis and graphs were made using GraphPad Prism 8.0.1 (GraphPad Software, San Diego, CA, USA).

## 3. Results

### 3.1. Development and Morphological Features of Breast Cancer Spheroids

**Figure 1:**
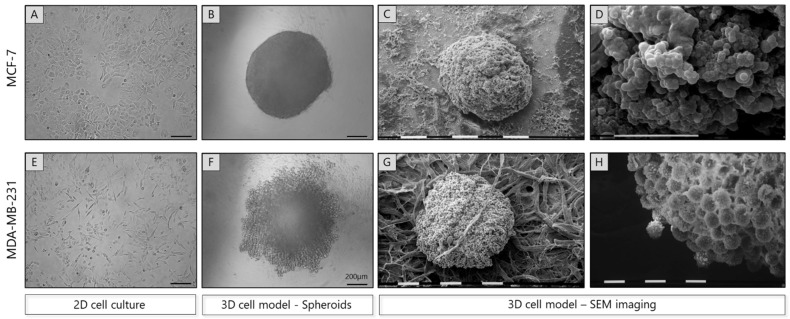
Morphological characterization of breast cancer spheroids, derived from two breast cancer cell lines with distinct ER profile and metastatic potential. Representative images of epithelial-like MCF-7 (**A**) and mesenchymal-like MDA-MB-231 (**E**) cells cultured in 2D plates as monolayers, as well as MCF-7 (**B**) and MDA-MB-231 (**F**) spheroids cultured for 72 h in U-shape, round bottom 96-well plates with ultra-low adhesive properties (scale bar, 200 μm). MCF-7 cells formed well-structured spheroids, while 3D MDA-MB-231 spheroids were relatively loosely organized in the outer layer, with a phenotypic transition to a globular, epithelial-like shape (**H**). SEM images of the MCF-7 (**C**,**D**), showing compacted cells and intercellular gaps of 5–10 µm, and MDA-MB-231 (**G**,**H**) spheroids, exhibiting superficial detaching cells, are also given [White/black scale bars, 0.1 mm (**C**,**G**) and 10 μm (**D**,**H**)].

To evaluate cancer cell properties mimicking the in vivo tumor growth and progression, we developed 3D cell culture models, particularly spheroids. To this end, MCF-7 and MDA-MB-231 breast cancer cells were cultured for 72 h in U-shape, round bottom 96-well ultra-low adhesive plates. Representative images, shown in Figure 1, illustrate the morphology of MCF-7 and MDA-MB-231 cells in 2D and 3D cultures, highlighting the differences in their organization under 3D conditions.

Notably, the less invasive, dome-structured MCF-7 cells (Figure 1A) form dense, well-structured spheroids (Figure 1B), indicative of their epithelial-like properties. In contrast, the more aggressive MDA-MB-231 cells (Figure 1E) form relatively loosely organized spheroids, particularly in the outer cell layer, which showed detaching and migrating cells from the spheroid surface, while maintaining a compact core (Figure 1F). It is worth noting that a phenotypic transition of the 2D cultured mesenchymal-like MDA-MB-231 cells (Figure 1E) into a profound globular, epithelial-like shape in 3D conditions (Figure 1F) was observed. The morphological properties of breast cancer cell spheroids were further validated using SEM, which provided additional confirmation of the phenotypic alterations and particular of the globular shape of MDA-MB-231 cells. Interestingly, as shown in Figure 1C,D, MCF-7 spheroids showed compacted cells which were individually indistinguishable, and developed intercellular gaps (5–10 µm wide), potentially to facilitate nutrient/O_2_ diffusion for cells into the inner portion of the spheroid. On the other hand, MDA-MB-231 spheroids, exhibiting more distinguishable cells with the superficial ones detaching from the spheroid surface, lack these gaps, likely allowing sufficient nutrient penetration through the intercellular spaces (Figure 1G,H).

### 3.2. MDA-MB-231 Cells Undergo Phenotypic Transition from 2D Culture to 3D Spheroids

The phenotypic transition observed in MDA-MB-231 cells during spheroid formation compared to their original elongated, mesenchymal-like morphology in 2D cultures, prompted us to further examine the expression levels of epithelial and mesenchymal markers in 2D and 3D cell models. E-cadherin and JAM-A were selected as representative epithelial markers (encoded by *CDH1* and *F11R* genes, respectively), while Slug and Vimentin were examined as mesenchymal markers (encoded by *SNAI2* and *VIM* genes, respectively).

**Figure 2:**
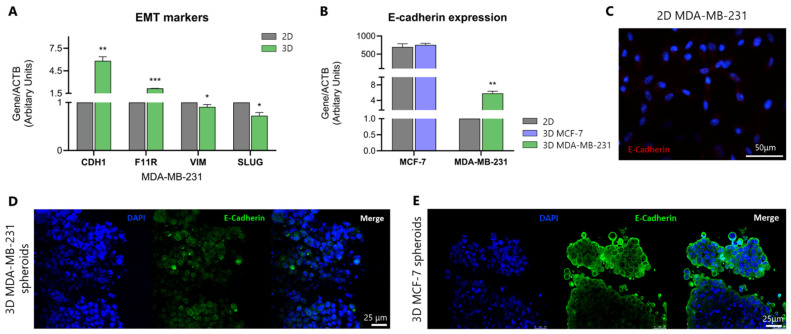
Three-Dimensional culture shifts EMT marker expression of MDA-231 cells towards an epithelial-like phenotype. Real-time qPCR analysis of *E-cadherin*/*CDH1*, *JAM-A*/*F11R*, *vimentin* and *SLUG* mRNA levels in 2D and 3D MDA-MB-231 cells (**A**). Comparative *E-cadherin* mRNA levels in MCF-7 and MDA-MB-231 cells, in 2D and 3D cell models (**B**). Protein expression levels of E-cadherin in MDA-MB-231 and MCF-7 spheroids are also indicated. Immunofluorescence imaging of E-cadherin in 2D cell culture (red) (**C**) and confocal microscopy of E-cadherin in 3D spheroids (green) in MDA-MB-231 (**D**) and MCF-7 (**E**) cells, respectively. Nuclei are shown in blue (DAPI). Scale bars are indicated. Each bar represents mean ± SD values from triplicate samples. One asterisk (*) indicates statistically significant differences (*p* ≤ 0.05), two asterisks (**) indicate statistically significant differences (*p* ≤ 0.01), while three asterisks (***) indicate statistically significant differences (*p* ≤ 0.001) compared to the control group (2D cell model).

Real-time qPCR analysis (Figure 2A) revealed a significant upregulation of the epithelial markers along with the concurrent downregulation of mesenchymal markers, indicating a shift from the mesenchymal-like morphology to a more epithelial-like phenotype. Specifically, a statistically significant increase in the epithelial markers *E-cadherin*/*CDH1* (3.8-fold change, *p* ≤ 0.001) and *JAM-A*/*F11R* (1.1-fold change, *p* ≤ 0.0001) gene expression levels was observed in MDA-MB-231 spheroids as compared to their levels in 2D cultures. This has been further confirmed by the decrease in the gene expression levels of the mesenchymal markers *SNAI2*/*SLUG* (0.3-fold change, *p* ≤ 0.001) and *VIM* (0.1-fold change, *p* ≤ 0.05) in spheroids as compared to 2D grown MDA-MB-231 cells. Nevertheless, the significantly increased *E-cadherin* expression in 3D MDA-MB-231 cells does not reach the levels found in the originally epithelial 2D MCF-7 cells (Figure 2B), highlighting the distinct phenotypic characteristics/differences observed in the two cell lines.

To further examine the protein expression levels of the epithelial marker E-cadherin in the MDA-MB-231 3D cell model, immunofluorescence staining combined with confocal microscopy were performed. The obtained data revealed upregulated E-cadherin protein levels in spheroids (Figure 2D) compared to 2D MDA-MB-231 cells, where E-cadherin expression was negligible (Figure 2C). As shown in Figure 2E, the protein expression levels of E-cadherin in epithelial-like MCF-7 spheroids provide a reference for the high E-cadherin levels, indicative of a strongly epithelial cell line. Taking into consideration the data above, it is clearly demonstrated that MDA-MB-231 cells shift to an epithelial-like phenotype when shifting from the 2D culture to 3D spheroids.

### 3.3. Spheroids’ Phenotypic Transitions Are Accompanied by Significant Alterations in the Expression of ERs, RTKs and Critical ECM Effectors

**Figure 3:**
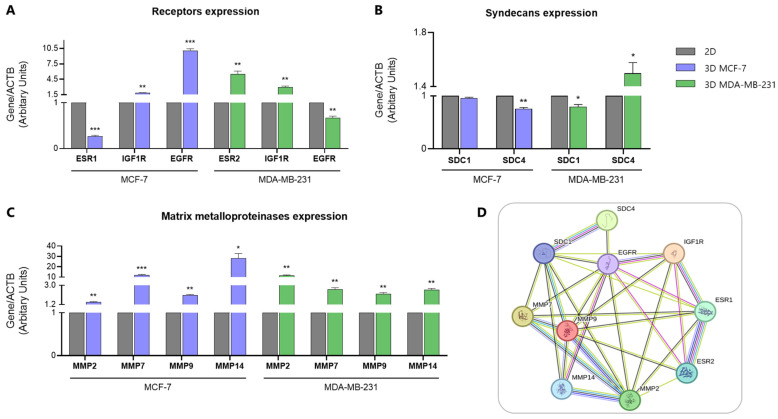
Gene expression levels of key signaling receptors and matrix effectors associated with breast cancer development and progression in MCF-7 and MDA-MB-231 spheroids compared to the respective 2D cell cultures. Real-time qPCR analysis of ERs and RTKs in 2D and 3D MCF-7 and MDA-MB-231 cells (**A**). Real-time qPCR analysis of MMPs in 2D and 3D MCF-7 and MDA-MB-231 cells compared to the respective 2D cell cultures (**B**). Real-time qPCR analysis of SDCs in 2D and 3D MCF-7 and MDA-MB-231 cells compared to the respective 2D cell cultures (**C**). Protein–protein interaction network constructed using the STRING database (**D**). Nodes represent individual proteins (gene names are given) and line colors indicate different interaction types. ESR1, ESR2, EGFR, IGF1R, SDC1, SDC4, MMP2, MMP7, MMP9 and MMP14 refer to ERα, ERβ, EGFR, IGF-IR, SDC-1, SDC-4, MMP-2, MMP-7, MMP-9 and MT1-MMP genes, respectively. Each bar represents mean ± SD values from triplicate samples. One asterisk (*) indicates statistically significant differences (*p* ≤ 0.05), two asterisks (**) indicate statistically significant differences (*p* ≤ 0.01), while three asterisks (***) indicate statistically significant differences (*p* ≤ 0.001) compared to the control group (2D cell model).

Following the development and morphological characterization of 3D spheroids, we first explored the expression of receptors, highly associated with breast cancer development and progression, in both 2D and 3D cultures. To this end, *ERs* (*ESR1*, *ESR2*), *IGF1R* and *EGFR* gene expression levels were evaluated. As shown in Figure 3A, following real-time qPCR analysis, we found a notable reduction in *ESR1* expression (0.7-fold change, *p* ≤ 0.001) in MCF-7 spheroids compared with 2D cultures, while *ESR2* expression levels increased dramatically (4.5-fold change, *p* ≤ 0.01) in MDA-MB-231 spheroids. *IGF1R* expression was increased in both 3D models (0.5-fold change, *p* ≤ 0.01 for MCF-7, and 2-fold change, *p* ≤ 0.01 for MDA-MB-231, respectively). In contrast, *EGFR* expression was significantly increased in 3D MCF-7 cells (9-fold change, *p* ≤ 0.001), but decreased in 3D MDA-MB-231 cells (0.3-fold change, *p* ≤ 0.01) compared to their respective 2D counterparts.

In respect to cell membrane PGs, *SDC1* expression a slight reduction was observed in 3D MCF-7 cells compared with 2D (not significant), while a more significant reduction (0.2-fold change, *p* ≤ 0.05) was observed in the 3D MDA-MB-231 cell model compared with 2D cells. Moreover, *SDC4* expression decreased in 3D MCF-7 cells (0.25-fold change, *p* ≤ 0.01) but increased in 3D MDA-MB-231 cells (0.5-fold change, *p* ≤ 0.05) (Figure 3B).

The expression levels of key matrix enzymes associated with migration and invasion during breast cancer progression were further evaluated. Real-time qPCR revealed significant differences in their expression between 2D and 3D cell models (Figure 3C). Particularly, all MMPs examined appeared upregulated in the 3D models compared to the respective 2D cells in both breast cancer cell lines. *MMP2* expression exhibited a noticeable increase in 3D MCF-7 cells (0.4-fold change, *p* ≤ 0.01), while a significantly greater upregulation was observed in the 3D MDA-MB-231 cell model (10-fold change, *p* ≤ 0.01) in comparison with the respective 2D cells. Moreover, a comparable 1-fold change increase in *MMP9* expression was observed in both 3D MCF-7 (*p* ≤ 0.01) and MDA-MB-231 (*p* ≤ 0.01) cell models vs. the 2D cultured cells. Furthermore, *MMP7* expression levels demonstrated a substantial increase in 3D MCF-7 (11-fold change, *p* ≤ 0.001) and 3D MDA-MB-231 cells (1.6-fold change, *p* ≤ 0.01), respectively. Finally, *MMP14*/*MT1-MMP* expression exhibited a dramatic increase in 3D MCF-7 cells (27-fold change, *p* ≤ 0.01) and 3D MDA-MB-231 cells (1.5-fold change, *p* ≤ 0.01). The data above further demonstrate that the transition of 2D cultured breast cancer cells to 3D is accompanied by significant alterations in the expression of receptors and matrix effectors implicated in breast cancer progression.

### 3.4. Protein–Protein Interaction Network Highlights the Relationship Between Key Receptors and Matrix Signatures

Taking into consideration the differential expression of certain ECM molecules and receptors in breast cancer cells, we further evaluated the relationship of ERs and the two RTKs, EGFR and IGF-IR, with critical matrix macromolecules implicated in breast cancer progression. To this end, a protein–protein interaction network was constructed using the STRING database [28]. To capture the distinct ER expression profiles of the two breast cancer cell lines employed, ESR1 (ERα) and ESR2 (ERβ) were incorporated into the network. EGFR and IGF1R, which drive signaling pathways involved in breast cancer development and progression, were also included due to their known crosstalk with E2-ER signaling pathways. Additionally, cell surface PGs (SDC1 and SDC4) and MMPs (MMP2, MMP7, MMP9, and MMP14) implicated in breast cancer progression were further included.

The constructed network (Figure 3D) highlights direct, experimentally determined interactions (purple lines) between receptors, such as ERα—ERβ, ERα—EGFR, ERα—IGF-IR, ERβ—EGFR, and ERβ—IGF-IR, as well as between SDC1—SDC4 and EGFR—MT1-MMP. These interactions underscore the crosstalk between ERs and RTKs, which act as critical players in breast cancer progression. Furthermore, the EGFR—MT1-MMP interaction suggests that EGFR signaling may regulate MT1-MMP activity, potentially influencing processes, such as cell migration and invasion.

The network also reveals strong co-expression (black lines) of EGFR with all matrix molecules examined, highlighting the central role of EGFR in regulating pathways affecting ECM composition within the breast TME. Co-expression patterns among MMPs further support their involvement in ECM remodeling, critical for tumor invasion and metastasis. It is worth noticing that the direct interaction between SDC1 and SDC4, alongside the co-expression of SDC1 with MMPs, suggest that cell surface PGs function as integral players in matrix remodeling, implying the role of SDCs in coordinating ECM dynamics. Conclusively, the protein–protein interaction network revealed a close relationship between the key receptors (ERs and RTKs) and matrix molecules associated with breast cancer.

### 3.5. Prognostic Values of ERs and Matrix Molecules in Luminal A and TNBC Patients’ Survival

**Figure 4:**
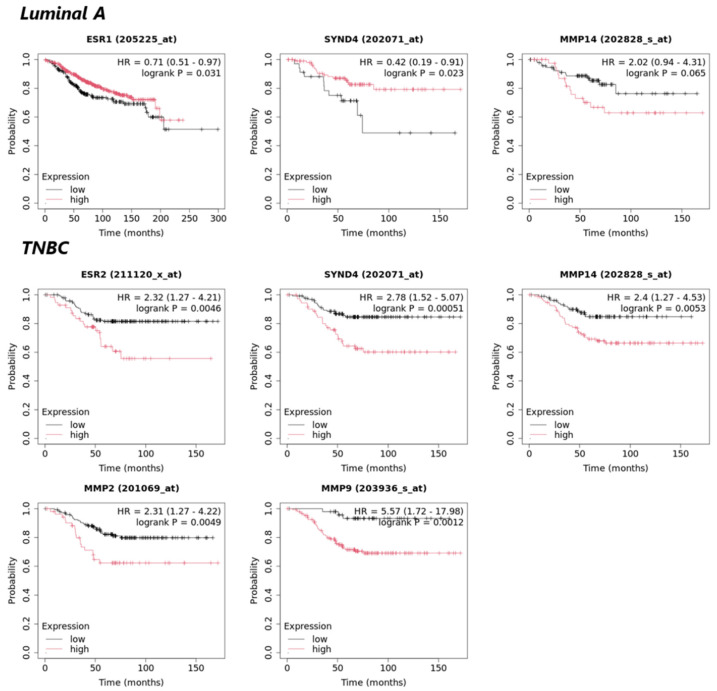
Meta-analysis of patients’ OS in different breast cancer patients’ datasets (Kaplan–Meier plots). *p* value and hazard ratio (HR) value were calculated using a log-rank test. *ESR1*, *ESR2*, *SYND4*, *MMP2*, *MMP9* and *MMP14* refer to *ERα*, *ERβ*, *SDC4*, *MMP2*, *MMP9* and *MT1-MMP*, respectively.

In order to further evaluate the relationship of the different expression profiles of ERs with matrix critical players and functional properties significantly affected upon transition from 2D cell monolayers to 3D spheroids, the prognostic values of ERs, SDC4 and MMPs, and their correlation with patients’ overall survival (OS) was further studied using the Kaplan–Meier plotter tool [27]. Results from Kaplan–Meier plotter (Figure 4) revealed that low expression of *ESR1* is significantly correlated with lower probability of OS in luminal A subtype patients’ (*p* ≤ 0.05). In contrast, in TNBC cases, the high expression of *ESR2* appears strongly associated with poorer prognosis and low OS probability (*p* ≤ 0.01).

Regarding the prognostic value of *SDC4*, results support that its low expression in Luminal A patients is linked to reduced patients’ OS probability (*p* ≤ 0.05). On the other hand, high expression of *SDC4* is shown to be significantly associated with poorer prognosis and lower OS probability in TNBC patients (*p* ≤ 0.001). Further Kaplan–Meier plotter results indicated that in both breast cancer subtype cases, the higher expression of *MMP14* showed a clear but not statistical trend for correlation with lower OS probability of patients’ OS (*p* = 0.065 in Luminal A and *p* = 0.053 in TNBC, respectively). Finally, results revealed that higher expression levels of *MMP2* and *MMP9* are significantly associated with poor outcomes and low OS probability of TNBC patients (*p* ≤ 0.01 and *p* ≤ 0.001, respectively).

### 3.6. Dissemination of Spheroids: A Model to Mimic the Initial Steps of Tumor Spreading

**Figure 5:**
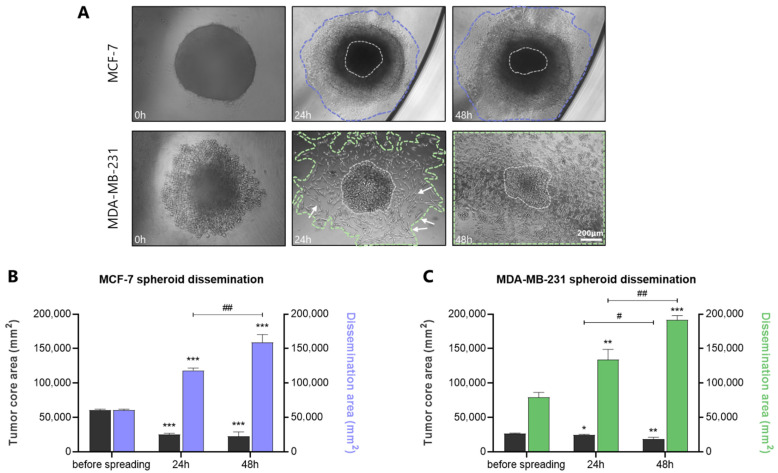
Dissemination of MCF-7 and MDA-MB-231 spheroids as a model to study the initial steps of tumor spreading/dissemination. Representative images of MCF-7 and MDA-MB-231 spheroids dissemination on polystyrene well plate at the timepoints of 0, 24, and 48 h (scale bar, 200 μm) (**A**). Quantification graph of the tumor core area and dissemination area of MCF-7 (**B**) and MDA-MB-231 (**C**) spheroids. Each bar represents mean ± SD values from three spheroids analysis per condition (*n* = 3). One asterisk (*) indicates statistically significant differences (*p* ≤ 0.05), two asterisks (**) indicate statistically significant differences (*p* ≤ 0.01) and three asterisks (***) indicate statistically significant differences (*p* ≤ 0.001) compared to the control group (2D cell model), while one hash (#) indicates statistically significant differences (*p* ≤ 0.05) and two hashes (##) indicate statistically significant differences (*p* ≤ 0.01) between 24 and 48 h intervals.

Cancer metastasis is the leading cause of cancer-related mortality, responsible for almost 90% of deaths [32]. To study tumor spreading and cancer cell dissemination, spheroids were transferred from the U-shape, round bottom 96-well plates with ultra-low adhesive properties to standard flat-bottom 96-well plates. Representative images (Figure 5A) show that, during dissemination (24–48 h), cells spread/migrate through the entire periphery of the spheroids, reverting to their 2D morphology. This phenotypic alteration is particularly pronounced in MDA-MB-231 cells, which even from 24 h, shift from the epithelial-like morphology observed in 3D spheroids to their “classical” 2D mesenchymal-like shape (indicated by arrows). It is worth noting that the highly aggressive MDA-MB-231 cells rapidly disseminate from the spheroid core, in comparison with the less invasive MCF-7 cells, which remain more confined. Notably, the core of both MCF-7 and MDA-MB-231 spheroids progressively diminishes in size after 24 h and 48 h, while the dissemination area significantly expands among the same intervals, highlighting the dynamic nature of tumor spreading (Figure 5B). By definition, the spheroid core refers to the innermost region of a multicellular spheroid, characterized by limited O_2_/nutrient availability, accumulation of metabolic waste, acidic conditions, and reduced cell proliferation/viability compared to the outer layers [33]. Quantification graphs of the spheroid core area and dissemination area of MCF-7 and MDA-MB-231 spheroids are further represented in Figure 5C.

**Figure 6:**
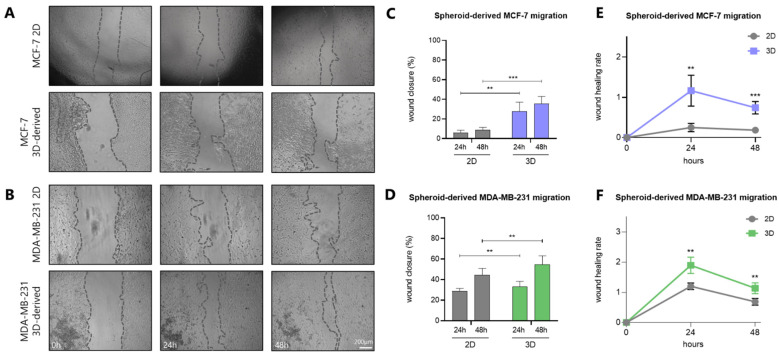
Spheroid-derived breast cancer cells migrate faster than the respective 2D cultured cells. Representative images of the wound healing assay of MCF-7 (**A**) and MDA-MB-231 (**B**) spheroid-derived cells at the time points of 0, 24 and 48 h (scale bar, 200 μm). Quantification graph of MCF-7 (**C**) and MDA-MB-231 (**D**) cell migration after 24 and 48 h. Wound healing rate of MCF-7 (**E**) and MDA-MB-231 (**F**) spheroid-derived cells after 24 and 48 h. Each bar represents mean ± SD values from triplicate samples. Two asterisks (**) indicate statistically significant differences (*p* ≤ 0.01), while three asterisks (***) indicate statistically significant differences (*p* ≤ 0.001) compared to the control group (2D cell model).

A key hallmark of cancer progression is the increased motility and invasiveness of cancer cells, which ultimately drive metastasis [34]. To this end, the migratory capacity of the two breast cancer cell lines was assessed under both 2D and 3D conditions. Notably, when allowed to spread to confluency in adherent tissue culture plates, spheroid-derived cells demonstrated significantly faster migration compared to their 2D counterparts of both cell lines, exhibiting a higher wound healing rate after 24 h and 48 h (Figure 6A,B). Specifically, 2D MCF-7 cells exhibited wound closure by *ca* 5% and 10% at 24 h and 48 h, respectively, whereas spheroid-derived MCF-7 cells achieved a *ca* 30% and 40% wound closure over the same time intervals (Figure 6C). As expected, a higher wound healing rate was noted in spheroid-derived MCF-7 cells in comparison with 2D cells (Figure 6E). In MDA-MB-231 cells, 2D cultured cells showed a *ca* 30% and 45% wound closure after 24 h and 48 h, respectively, while spheroid-derived cells reached a *ca* 35% and 55% wound closure over the same time points (Figure 6D), again with a higher wound healing rate in 3D-derived cells vs. 2D monolayers (Figure 6F).

## 4. Discussion

In this study, we aimed to develop and characterize 3D breast cancer cell-derived spheroids using a matrix-free platform, allowing for a comparative analysis with conventional 2D cell cultures. Spheroids are considered as a relatively simple yet powerful 3D cell model, that more accurately replicates the growth of solid tumors in three dimensions, the avascular TME during cancer development and could also be used to study the initial steps of cancer dissemination [35,36].

Cancer cells grown in 3D models exhibit alterations in their gene expression profiles compared to cells in 2D monolayers across various solid tumors, including breast cancer [5]. During tumor spheroid formation, cells initially aggregate through loose integrin-ECM interactions, while over time, they establish tighter cell–cell contacts, primarily mediated by increased E-cadherin expression and reduced N-cadherin levels [37]. Notably, MDA-MB-231 3D spheroids cultured within hydrogel bioscaffold exhibit positive E-cadherin staining, further supporting this transition [38]. Our findings on EMT markers expression align with aforementioned studies in other solid tumors (e.g., see Table 1 in [5]), further supporting the relevance of the matrix-free platform. Regarding MCF-7 spheroids morphology, Han et al. (2021) have discussed that ERα^+^ breast cancer cell lines are characterized by the formation of dense and compact spheroids, corroborating our findings [37]. Moreover, Madhavan et al. (2023) have previously demonstrated that T47D-derived (Luminal A) spheroids exhibit intercellular spaces predominantly in the outer regions, whereas the inner regions become progressively compact with reduced spacing [39]. This spatial heterogeneity aligns with our observations in MCF-7 spheroids–characterized by tight cell–cell contacts and strong E-cadherin expression–and potentially supports the hypothesis that peripheral intercellular gaps may aid O_2_/nutrient diffusion toward the spheroid core. In contrast, spheroids derived from MDA-MB-231 cells, which exhibit lower E-cadherin expression and reduced intercellular adhesion junctions, did not display such features. Though, the role of intercellular gaps within spheroids remains to be functionally validated.

As mentioned before, ERs comprise key players in breast cancer development and progression. Their expression profile appears upregulated in thyroid cancer cell spheroids vs. respective 2D cultures [40]. However, in our study, *ESR1* levels were significantly downregulated in MCF-7 spheroids compared to their 2D counterparts, while *ESR2* levels appeared highly upregulated in 3D MDA-MB-231 cells relative to 2D cultures. Although no experimental data confirm the downregulation of ERα in cancer cell spheroids, our research group has previously shown that ERβ potentially promotes the aggressive phenotype of TNBC, also in in vivo models [41]. Kaplan–Meier plotter database analyses further associate these receptors expression patterns with poorer patient OS probability, further highlighting the clinical relevance of *ESR1* and *ESR2* expression in Luminal A and TNBC patient outcomes, respectively (*p* ≤ 0.05 and *p* ≤ 0.05). The fact that these two ERs exhibit distinct distribution patterns across tissues and cell subpopulations suggests that their functions may be dependent on cancer type and cellular context [40].

In respect to RTKs, data from THPA indicate that *IGF1R* is highly expressed in MCF-7 cells (*n*TPM = 52.8), whereas *EGFR* is the predominant receptor in MDA-MB-231 cells (*n*TPM = 61.6) [42]. The dual role of IGF-IR in breast cancer progression has been extensively documented. Notably, multiple studies have shown a strong correlation between the expression of IGF-IR and the luminal-type tumors, along with its association with improved breast cancer-specific survival in patients. Furthermore, high IGF-IR expression is closely linked to hormone receptor-positive breast cancer and is associated with a favorable prognosis [43,44]. Our results demonstrated that *IGF1R* levels were elevated in MCF-7 spheroids compared to their 2D counterparts, validating the physiological relevance of the 3D model used. On the other hand, our study showed decreased *EGFR* levels in MDA-MB-231 spheroids compared to the respective monolayer 2D cultures. Previous studies have reported the downregulated EGFR in 3D cells in hormone-dependent breast cancer cells [45,46], but also in pancreatic ductal adenocarcinoma 3D cell cultures [47]. Conversely, other studies have demonstrated the upregulation of EGFR and other human epidermal growth factor receptor family members in 3D breast cancer cells cultured in poly-HEMA- or Matrigel^®^- coated plates [48,49]. Taking into consideration that clinical studies have associated EGFR overexpression with poor prognosis and unfavorable outcomes in highly aggressive TNBC patients [50], it is, therefore, important to highlight that culture conditions can possibly influence EGFR expression differently, suggesting a context-dependent regulation of this receptor.

SDC1 and SDC4 have been associated with cell signaling and functional properties, while also holding prognostic value in various cancer types [22]. In breast cancer, *SDC1* can act as either a favorable or unfavorable prognostic factor, depending on the hormonal status and cancer subtype [51]. In our study, *SDC1* levels appeared statistically significantly decreased only in 3D MDA-MB-231 cells in comparison with their 2D counterparts. Our results could be attributed to the altered cell–cell contacts and ECM deposition between different spheroid culture techniques. Interestingly, previous studies have demonstrated that SDC1 expression is not only limited to cancer cells, but also occurs in the stromal compartment across various tumors, particularly in invasive breast cancer, supporting the importance of interactions between different cell types within the TME [52]. Therefore, in spheroids composed only of cancer cells, the absence of stromal interactions could potentially reduce *SDC1* expression.

Regarding *SDC4* expression, our results revealed a decrease in 3D MCF-7 spheroids, whereas an increase in MDA-MB-231 spheroids was observed in comparison to monolayer cultures. This result is also supported by data from the Kaplan–Meier plotter database, which further indicate that lower *SDC4* expression in Luminal A patients is correlated with low OS probability (*p* ≤ 0.05). On the other hand, high SDC4 expression appears significantly associated with poorer prognosis and lower OS probability in TNBC patients (*p* ≤ 0.001). These findings demonstrate that *SDC4* may exhibit a subtype-dependent prognostic role in breast cancer, acting both as a tumor suppressor and tumor progression marker depending on breast cancer subtype [53]. Literature has revealed that high expression of SDC4 is an unfavorable biomarker for various cancer types, including ER-negative breast cancer [54]. Afratis et al. (2017) further showed that SDC4 expression is downregulated in MCF-7 SP10^+^ (ERα^−^) cells, suggesting that ERα activity is linked to SDC4 regulation and contributes to the aggressiveness of MCF-7 cells [55]. Thus, the co-downregulation of *ESR1* and *SDC4* observed in our 3D MCF-7 model likely reflects a shift toward a more aggressive phenotype.

The biological roles of MMPs and their pharmacological targeting in cancer have been thoroughly revised in the literature [56,57]. MMP2/7/9 and MT1-MMP appear co-expressed from the protein interaction network constructed by STRING database and are known to hold key roles in breast cancer development and progression [57]. Particularly, gelatinases MMP2 and MMP9 appear highly expressed in breast cancer tissues and are strongly associated with tumor staging and metastasis, particularly driving TNBC metastasis, thereby serving as important biomarkers for assessing prognosis and guiding treatment strategies [58,59]. It has been previously shown that MMP2 and MMP9 are upregulated by hypoxia in breast and colon cancer cells via a HIF1-dependent mechanism [60]. In this study we demonstrated that *MMP2* and *MMP9* expression appears significantly increased in the 3D cell models compared to the respective 2D cells. Notably, Kaplan–Meier plotter analysis also revealed that high *MMP2* and *MMP9* expression is significantly associated with poorer prognosis and lower OS probability of TNBC patients (*p* ≤ 0.005 and *p* ≤ 0.001, respectively), further supporting the prognostic value of gelatinases in TNBC outcomes. The upregulation of gelatinases is further evaluated in 3D breast cancer models derived from the less-metastatic shERβ MDA-MB-231 and the TNBC Hs578T cells [26]. However, we should note that *MMP2* and *MMP9* expression (data derived from THPA) is minimal in 2D cell cultures of both MCF-7 and MDA-MB-231 cells, suggesting that those cells do not heavily rely on these proteases for matrix remodeling.

Our results further indicated substantially upregulated *MMP7* in 3D MCF-7 and MDA-MB-231 cells in comparison with the respective 2D counterparts. *MMP7* gene functions as a proto-oncogene, driving breast cancer development and progression [61]. Wang et al. have previously shown that MMP-7 overexpression in MCF-7 cells enhances cell invasion and metastasis [62]. Although MMP-7 expression has not been really associated with prognosis or outcomes of Luminal A breast cancer patients, our findings probably suggest that MCF-7 cells grown in 3D models undergo a transition towards a more invasive phenotype. As it has been previously shown from our research group, *MMP7* appears upregulated in 3D breast cancer spheroids (derived from shERβ MDA-MB-231 and Hs578T cells, respectively) [26].

Finally, our study resulted in a significant increase in *MMP14* levels in the 3D cell models of both cell lines compared to the 2D cells. Numerous studies support that high MT1-MMP is associated with various cancer types development and progression [63]. Carey et al. (2017) have previously demonstrated that MCF-10A normal mammary cells cultured in a 3D collagen bioscaffold exhibited higher MT1-MMP gene expression compared to those cultured in 2D monolayers [64]. Additionally, MT1-MMP is regulated by hypoxia-inducible factors (HIF-1α and HIF-2α), particularly upregulated under hypoxic conditions, supporting the cellular adaptation to low oxygen levels within the spheroid core [65]. To conclude, Kaplan–Meier plotter analyses indicated that the higher expression of *MMP14* was associated with lower patients OS probability in both Luminal A and TNBC patients. All this evidence suggests that *MMP14*/MT1-MMP could be regarded as a key marker of breast cancer progression, with its elevated expression in 3D models compared to 2D likely being influenced by the highly hypoxic conditions within the spheroid core. Furthermore, the upregulation of proteolytic enzymes under 3D culture conditions may contribute to ectodomain shedding of some of the receptors analyzed in our study (*SDC1, SDC4, EGFR*), which may further modulate their function [22].

Cancer cells’ functional properties, such as dissemination and migration, were also involved in order to evaluate the cell models in preclinical studies. In vitro models for studying cancer cell migration and invasion primarily include transwell migration assays, wound healing assays, and time-lapse cell tracking in 2D monolayer cultures, to assess cellular motility; however, these models fail to accurately capture the EMT process [5,35]. In this experimental study, both MCF-7 and MDA-MB-231 cells were able to adhere and spread over time intervals upon transferring spheroids in polystyrene 2D culture plates in multiple directions. Breast cancer cell dissemination in multiple directions across the entire periphery of the spheroids, along with the significant cell phenotypic reversion to their typical 2D morphology, serves as a model to mimic collective cell migration during EMT, invasion, and ultimately, metastasis. Studies have already demonstrated that spheroid-derived MDA-MB-231 cells exhibit higher migratory capacity than the respective 2D cultured cells after 24 h, whether the spheroids are cultured in a bioscaffold-free platform or within a gelatin bioscaffold [66,67]. Other studies have also shown that spheroid-derived MCF-7 cells (i.e., MCF-7 spheroids were developed within a modified chitosan hydrogel) migrate faster than the respective 2D cells [68]. Our results further validate previous studies where the same cell lines were utilized, with the difference that in the present study spheroids were cultured in a matrix-free model.

The observed upregulation of MMPs in the 3D breast cancer model is consistent with the enhanced migratory capacity of cancer cells compared to 2D cultures. MMPs hold a pivotal role in matrix remodeling, thereby facilitating cellular detachment and invasion—processes that are critical for cancer dissemination [57]. This increase in MMP expression likely reflects an adaptive response to the spatial and mechanical constraints of the microenvironment, where cell-ECM interactions promote migratory signaling. Within this context, dynamic cell–cell and cell–matrix interactions potentially stimulate signaling cascades that drive cytoskeletal reorganization and motility, thereby promoting collective migration and cancer cell dissemination [69]. In parallel, the observed alterations in the expression levels of RTKs and SDCs (which act as crucial co-receptors and modulators of growth factor signaling) further support the hypothesis of a reprogrammed intercellular signaling that alters the communication between cancer cells and their surrounding matrix [22,24]. Collectively, these findings indicate that the 3D culture platform induces not only structural and behavioral adaptations, but also profound molecular changes that favor invasive signaling, underscoring its physiological relevance as a model for studying breast cancer progression and therapeutic responses.

## 5. Conclusions

**Figure 7:**
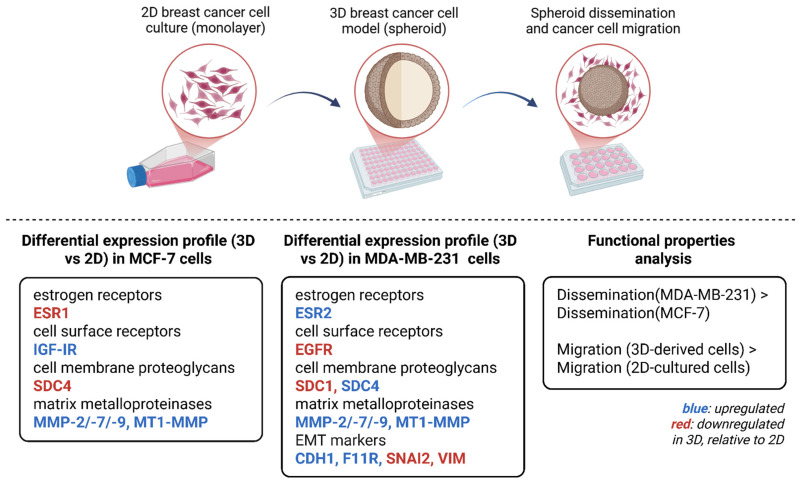
Schematic representation of the main results obtained using 3D vs. Two-dimensional cell cultures in terms of morphological and functional cell properties and key matrix effectors expression patterns. Significant morphological differences were noted upon spheroid formation utilizing breast cancer cell lines with distinct ER expression status, MCF-7 and MDA-MB-231. Upon spheroid dissemination, a collective migration (cell dissemination) was noted throughout the periphery of the spheroids, while an increased dissemination area was observed in MDA-MB-231 cells, compared to MCF-7 cells. Finally, spheroid-derived cells exhibited faster migration in comparison to the migratory capacity of the respective 2D-cultured cells. Furthermore, significant alterations in the expression levels of critical EMT markers (CDH1, SNAI2, VIM), ERs and cell surface receptors (EGFR, IGF-IR), as well as major ECM components (SDCs, MMPs) implicated in breast cancer progression were observed, in comparison to their respective 2D counterparts (blue: upregulated; red: downregulated relative to 2D). The prognostic values of ERs and matrix molecules in luminal A and TNBC patients’ overall survival probability was further examined, confirming the reliability of the model. Created in BioRender.com.

Conclusively, traditional 2D monolayers present significant limitations in replicating the complex architecture of in vivo solid tumors, which is essential for accurately studying cancer initiation, progression, and metastasis. The main results of our study are summarized in Figure 7. The obtained data showed notable alterations in the formation and functional properties of breast cancer cell-derived spheroids, which were also accompanied by significant alterations in the expression levels of several receptors, ECM signatures, as well as EMT markers in comparison with the respective 2D cell cultures. Data derived from bioinformatic tools further demonstrate that 3D spheroids appear to be a more representative model for studying the molecular and functional properties of solid tumors, as well as for pharmaceutical targeting. Further studies are warranted to elucidate the intricate cell–matrix interactions, as well as the epigenetic regulatory mechanisms that govern cellular behavior within 3D spheroid models. Incorporating physiologically relevant matrices that mimic the breast TME, as well as including patient-derived spheroid cultures would allow for a more accurate representation of cell–matrix interactions, which are crucial for regulating cell signaling, migration, invasion, as well as therapeutic responses.

## Figures and Tables

**Table 1:** Primer sequences used for real-time polymerase chain reaction analysis.

Gene		Primer Sequence (5′-3′)	Annealing T (°C)
*ESR1*	F	TGATGAAAGGTGGGATACGA	60
	R	AAGGTTGGCAGCTCTCATGT	
*ESR2*	F	TCCATGCGCCTGGCTAAC	60
	R	CAGATGTTCCATGCCCTTGTTA	
*EGFR*	F	ATGCTCTACAACCCCACCAC	60
	R	GCCCTTCGCACTTCTTACAC	
*IGF1R*	F	ACGAGTGGAGAAATCTGCGG	60
	R	ATGTGGAGGTAGCCCTCGAT	
*CDH1*	F	TACGCCTGGGACTCCACCTA	60
	R	CCAGAAACGGAGGCCTGAT	
*F11R*	F	CCGTCCTTGTAACCCTGATT	60
	R	CTCCTTCACTTCGGGCACTA	
*VIM*	F	GGCTCGTCACCTTCGTGAAT	60
	R	GAGAAATCCTGCTCTCCTCGC	
*SNAI2*	F	AGACCCTGGTTGCTTCAAGGA	60
	R	CTCAGATTTGACCTGTCTGCAAA	
*SDC1*	F	AGGACGAAGGCAGCTACTCCT	60
	R	TTTGGTGGGCTTCTGGTAGG	
*SDC4*	F	GTGTCCAACAAGGTGTCAATGT	60
	R	CGGTACATGAGCAGTAGGATCA	
*MMP2*	F	CGTCTGTCCCAGGATGACATC	62
	R	ATGTCAGGAGAGGCCCCATA	
*MMP7*	F	GCTGGCTCATGCCTTTGC	62
	R	TCCTCATCGAAGTGAGCATCTC	
*MMP9*	F	TTCCAGTACCGAGAGAAAGCCTAT	62
	R	GGTCACGTAGCCCACTTGGT	
*MMP14*	F	CATGGGCAGCGATGAAGTCT	60
	R	CCAGTATTTGTTCCCCTTGTAGAAGTA	
*ACTB*	F	TCAAGATCATTGCTCCTCCTGAG	60
	R	ACATCTGCTGGAAGGTGGACA	

## Data Availability

The raw data supporting the conclusions of this article will be made available by the corresponding authors on request.

## Abbreviations

The following abbreviations are used in this manuscript:

2D/3D	two-dimensional/three-dimensional
E2	17β-estradiol
ECM	extracellular matrix
EGFR	epidermal growth factor receptor
EMT	epithelial-to-mesenchymal transition
ERα/ERβ	estrogen receptor alpha/estrogen receptor beta
GFs	growth factors
HER2	human epidermal growth factor receptor 2
IGF-IR	insulin-like growth factor receptor
JAMs	junctional adhesion molecules
miRNAs	microRNAs
MMPs	matrix metalloproteinases
nTPM	number of transcripts per million
OS	overall survival
PGs	proteoglycans
PR	progesterone receptor
RTKs	receptor tyrosine kinases
SDCs	syndecans
SEM	scanning electron microscopy
TME	tumor microenvironment
TNBC	triple-negative breast cancer
